# Selective Immune Clearance and Lung Repair in Pulmonary Fibrosis

**DOI:** 10.3390/biology15211991

**Published:** 2026-11-03

**Authors:** Mingwei Sima, Zhengyuan Fang, Jiayu Liu, Yan Cui, Yongshi Liu, Jingjing Liu, Yan Bi, Donghui Yue

**Affiliations:** 1 College of Traditional Chinese Medicine, Changchun University of Chinese Medicine, Changchun 130117, China; smmwyjs@163.com (M.S.); 18804310249@163.com (J.L.); 2 College of Basic Medicine, Changchun University of Chinese Medicine, Changchun 130117, China; fangzhengyuan94@163.com (Z.F.); 13611371417@139.com (J.L.); cycczyydx@163.com (Y.C.); lys1234561014@163.com (Y.L.); 3 College of Continuing Education, Changchun University of Chinese Medicine, Changchun 130117, China

**Keywords:** pulmonary fibrosis, idiopathic pulmonary fibrosis, immune surveillance, cellular senescence, immunopeptidomics, spatial biology, chimeric antigen receptor, alveolar regeneration

## Abstract

Selective immune clearance could limit pulmonary fibrosis by removing disease-sustaining cells, but its value depends on preserving the capacity of the remaining tissue to repair. This narrative review examines PubMed literature searched through 10 October 2026, prioritizing studies published from 10 October 2024 to 10 October 2026 while retaining relevant earlier investigations. We compare human tissue profiling, immunopeptidomics, cell assays and animal interventions according to target recognition, effector-dependent elimination, treatment phase and retained repair capacity. Vaccination, checkpoint blockade and engineered immune cells provide different combinations of cytotoxicity, altered cell abundance and antifibrotic activity. However, repair-associated senescence and conflicting FAP-targeting outcomes show why marker expression alone cannot determine which cells should be removed. Epithelial proliferation and inferred cell trajectories are therefore assessed separately from lineage-supported differentiation, alveolar organization and sustained barrier, vascular and physiological recovery. The available studies do not yet establish that selective clearance produces lasting alveolar recovery, and clinical benefit in IPF remains unproven. Functional target validation, treatment after fibrosis is established, and follow-up after treatment withdrawal are priorities for translation.

## Simple Summary

Pulmonary fibrosis replaces portions of the lung’s gas-exchange surface with scar tissue. Immune targeting could remove cells that sustain this process, but some candidate targets are also present in cells needed for repair. This review therefore compares the cells targeted, the evidence for their removal, treatment timing and the repair capacity of the remaining tissue. Vaccines, antibodies and engineered immune cells have reduced experimental fibrosis in some settings, whereas other interventions have impaired matrix processing or damaged useful cells. These differences make cell function and disease stage relevant to treatment selection. We distinguish partial responses, such as reduced collagen or increased epithelial proliferation, from recovery of organized alveoli and sustained lung function. These findings support assessing whether immune targeting can remove disease-sustaining cells while preserving the lung’s capacity for repair. However, whether these approaches provide sustained clinical benefit in patients with pulmonary fibrosis remains to be established.

## 1. Introduction

Current antifibrotic treatments show that disease progression can be slowed, but how damaged lung tissue might recover remains unresolved. Idiopathic pulmonary fibrosis (IPF) is a specific diagnosis involving epithelial injury, abnormal repair and matrix accumulation; progressive pulmonary fibrosis (PPF) describes progression in fibrotic interstitial lung diseases other than IPF [1]. Nintedanib and pirfenidone slowed lung-function decline in randomized trials, and the 2025 FIBRONEER-IPF trial likewise reported a smaller decline in forced vital capacity with nerandomilast than with placebo [2,3,4]. These benefits establish slower functional deterioration, without demonstrating reconstruction of scarred alveoli.

Selective immune clearance refers to immune-effector-dependent physical elimination of a defined target-cell population. By contrast, functional modulation can suppress a pathogenic activity without removing the cell. This distinction matters because fibroblasts and epithelial intermediates may sustain fibrosis while retaining functions needed for repair. The consequences of immune targeting therefore depend both on what happens to the target and on which functions remain in the tissue.

The central question is how immune targeting can remove cells that sustain disease while preserving the ability of the lung to repair. Reviews of senescence in IPF, immune surveillance, fibroblast regulation of repair and pulmonary CAR-T therapy describe the relevant biological processes [5,6,7,8]. Here, we compare these approaches according to the evidence for recognition or elimination, the phase at which treatment begins and the effects on reparative cells and tissue function. Vaccination schedules, HLA-E/NKG2A experiments and conflicting FAP-targeting results illustrate why similar targeting concepts can produce different outcomes. This comparison identifies the evidence needed to connect an antifibrotic response to sustained recovery.

**Figure 1:**
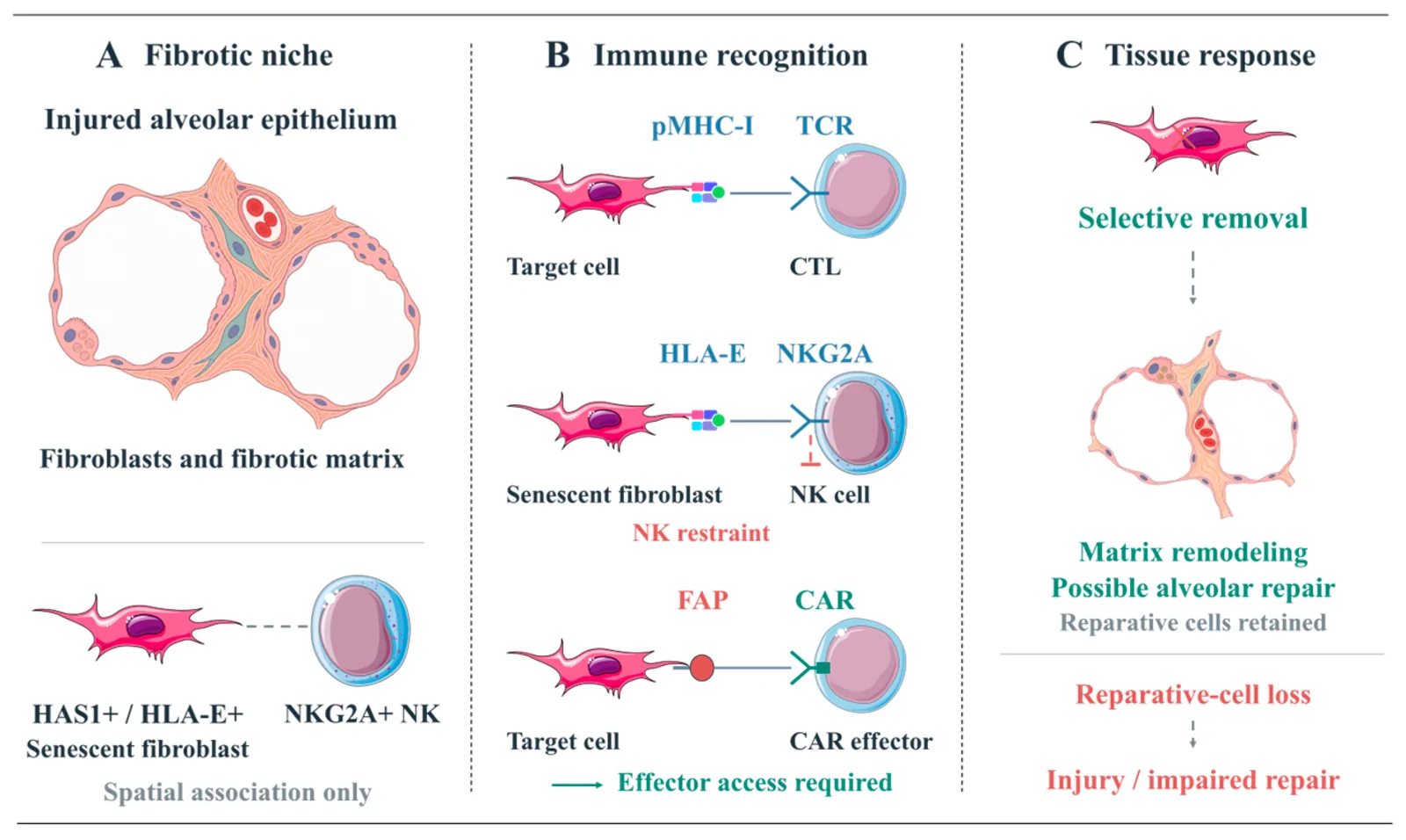
Cell targets and tissue responses to immune clearance in pulmonary fibrosis. (**A**) Injured epithelium, fibroblasts and immune cells occupy heterogeneous fibrotic regions. The proximity of HLA-E-positive senescent HAS1 fibroblasts to NKG2A-positive natural killer (NK) cells represents human spatial association, not proof of functional inhibition in that individual contact. (**B**) Peptide presentation, inhibitory receptors and surface antigens provide distinct recognition mechanisms; effector access and target-cell fate require separate tests. (**C**) Matrix remodeling and alveolar repair are possible responses after selective removal, whereas loss of reparative cells may worsen injury. Dashed arrows indicate proposed tissue outcomes, not a demonstrated sequence of clearance followed by regeneration. The schematic summarizes evidence from different experimental systems [9,10,11,12,13,14,15,16]. Selected cell icons were adapted from Servier Medical Art (CC BY 3.0), and MHC-I symbols from Helicase 11 (CC BY 4.0), via Bioicons. Icons were resized and assembled for this schematic; the original domain labels on the MHC-I symbol were removed and its orientation adjusted.

We first examine which disease-associated cell states may warrant elimination and which should be preserved. Antigen presentation and checkpoint signaling are then related to effector access, treatment timing and the measured response to vaccines, endogenous immune cells and engineered effectors. The later sections evaluate matrix remodeling, epithelial differentiation and pulmonary safety. Throughout, human associations, functional cell experiments and animal outcomes are kept distinct; Figure 1 outlines how target selection and the response of the remaining tissue are connected.

## 2. Literature Search and Evidence Assessment

This narrative review examined immune targeting and tissue repair in pulmonary fibrosis. PubMed was systematically searched from May 2026 through 10 October 2026 using Medical Subject Headings and title/abstract terms for pulmonary fibrosis combined with immune recognition, senescence, checkpoint signaling, engineered immune cells and alveolar repair. Targeted source checks and reference tracing identified additional mechanistic studies of cell niches, matrix mechanics and senescence-associated immune recognition in other organs. Searches covered publications dated 1 January 1900 through 10 October 2026, prioritizing studies published from 10 October 2024 through 10 October 2026. No language or species filters were applied to the primary searches. The principal search strategies, publication-date restrictions and retrieval limits are presented in Supplementary Material S1 (Table S1); the selection criteria are summarized in Table S2.

Titles and abstracts were assessed for relevance to target identity, immune recognition, effector-dependent clearance, treatment timing or repair. Potentially informative reports were examined in full text, including relevant methods, results, figures and available supplements. Priority was given to original experiments that tested a mechanism, compared treatment phases, documented adverse or conflicting outcomes, or measured reparative function. Earlier studies were retained for foundational causal experiments, lineage tracing, important counterexamples and clinical safety. Human IPF findings were distinguished from studies in other diseases, organs or experimental models. Reviews and guidelines provided context and additional references; original studies were used to support experimental conclusions.

The initial relevance-selected reference collection contained 191 unique records. The final bibliography includes 139 of these records and 16 additional sources identified through targeted assessment or reference tracing, giving 155 references. The remaining 52 candidates were not selected for citation. The selection process and reference counts are summarized in Supplementary Material S1 (Table S3), and individually retrieved supporting sources included in the initial collection are listed in Table S4.

Findings were synthesized qualitatively by experimental system, target, effector mechanism, treatment phase and measured outcome. Association or antigen expression, functional recognition or modulation, physical cell elimination and tissue recovery were considered separately. Immune-mediated elimination required loss of a defined target population supported by death or engulfment measurements and a test of effector dependence. Marker loss, altered secretion and soluble-tracer uptake alone were not treated as proof of elimination. Collagen reduction was also distinguished from epithelial differentiation, restored alveolar structure and sustained physiological improvement. ARRIVE 2.0 and the American Thoracic Society workshop informed the interpretation of animal experiments [17,18]. No meta-analysis or formal risk-of-bias scoring was performed.

## 3. Cell States in Fibrosis and Repair

### 3.1. Epithelial and Stromal Populations

Selective clearance requires distinguishing cells that maintain disease from those needed for repair. Human tissue maps provide a starting point by resolving heterogeneity within broad epithelial and stromal categories. Single-cell studies identified mixed alveolar and airway epithelial identities [19] and spatially distinct mesenchymal populations [20]. Complementary analyses identified matrix-associated basal cells and POSTN/CTHRC1-expressing fibroblasts, while spatial profiling localized K13-expressing epithelium to remodeled distal airways and honeycomb cysts [21,22]. These findings describe anatomically organized, heterogeneous lesions rather than a uniformly abnormal cell population. Although cell identity and location can guide target selection, the effect of depleting these populations on lung function must still be tested.

Functional experiments strengthen this rationale when they link mapped populations to processes that sustain injury. CTHRC1-high fibroblasts and aberrant transitional epithelium occupied shared neighborhoods, and combined transforming growth factor-β (TGF-β), interleukin-1β (IL-1β) and tumor necrosis factor-α (TNF-α) induced maladaptive differentiation in organoids [23]. WNT5A-positive/CTHRC1-positive myofibroblasts were also present in fibroblastic foci and dense scars. PAK inhibition reduced experimental fibrosis, although the contribution of PAK2 was not isolated in vivo [24]. These observations link lesion-associated states to processes that can be modified experimentally. However, susceptibility to intervention also varies with disease context. Fibroblasts from organizing pneumonia, connective-tissue-disease-associated interstitial lung disease and IPF differed in susceptibility to apoptosis [25]. Shared collagen accumulation therefore does not imply identical targets or sensitivity to clearance.

Within the same lesion, immune contacts may sustain injury or help the surviving epithelium recover. Imaging mass cytometry-associated CD206-high macrophages with alveolar basal intermediates across sampled IPF regions [26], while airway brushings from newly diagnosed patients showed altered macrophage populations and inferred epithelial–immune interactions [27]. These observations place epithelial and stromal states in their immune context, but they do not establish signaling direction or how individual lesions evolve. A candidate target should therefore be characterized by its state and local contacts, followed by functional perturbation to determine whether it drives injury or supports surviving epithelium. This is the step needed to translate spatial mapping into a rationale for selective treatment.

### 3.2. Fibroblast Plasticity and Repair

Activated fibroblasts are heterogeneous treatment targets because matrix production can coexist with support for epithelial recovery. Mouse lineage tracing and single-cell analysis demonstrated reversible transitions between lipofibroblasts and Cthrc1-positive myofibroblasts during injury and resolution [28]. Aging limited this recovery, with delayed resolution and weaker support for alveolar type 2 (AT2) growth, although parts of the differentiation program persisted [29]. Persistent alveolar-fibroblast-derived ADAMTS4-positive cells were also identified during delayed resolution, with supporting observations in human tissue and ex vivo systems [30]. Thus, activation alone does not describe the properties relevant to targeting; reversibility and persistence also matter. Treatments that suppress persistent fibrogenic activity should therefore assess the repair capacity retained by the remaining stroma.

Comparing injury-associated profiles with behavior during repair makes this distinction clearer. Col1a1-, Plin2- and Acta2-labeled cells converged toward fibrotic profiles after bleomycin but diverged during repair [31]. A similar profile during injury therefore need not imply a similar contribution to resolution. Functional experiments provide complementary evidence. Resolving fibroblast populations supported epithelial recovery in mouse-transfer experiments and experimentally fibrosed human lung slices [9], whereas mesenchymal Atg5 deletion reduced secretion of repair-associated factors and impaired AT2 proliferation and differentiation [32]. Although these experiments address different stromal functions, both make epithelial support relevant to treatment assessment. To determine whether tissue is recovering, a reduction in fibroblast abundance should be evaluated together with epithelial support and matrix turnover.

The relevance of senescence to depletion depends on the injury phase and the function of the marked cells. In healthy mouse lungs, p16INK4a-positive fibroblasts adjacent to epithelial progenitors increased epiregulin secretion after inflammatory injury. Senolytic treatment or fibroblast p16 deletion impaired club-cell recovery after naphthalene exposure [11]. This is an airway-repair experiment, not evidence that these cells restore alveoli in human IPF. Similarly, HAS2 experiments linked increased fibroblast senescence to reduced fibrotic activity [33]. Thus, a transient injury-associated response should be distinguished from a persistent population whose secretory activity sustains disease. Cell identity, anatomical location, persistence and effects on neighboring epithelium should guide the choice among preserving, modulating and eliminating a senescence-associated population.

### 3.3. Alveolar Epithelial Differentiation

For epithelial targets, persistence and completed differentiation distinguish a repair intermediate from a potentially harmful state. Mouse lineage tracing identified Krt8-positive transitional and pre-AT1 populations with DNA-damage, TP53 and senescence-associated programs during AT2-to-AT1 differentiation [34,35]. Related epithelial states accumulate in human fibrotic lungs. Human KRT5-negative/KRT17-positive populations have been linked to these mouse intermediates by cross-species transcriptomic analysis, but their differentiation paths were inferred rather than lineage traced [36]. We therefore use Krt8-positive transitional cells for the experimentally traced mouse populations and KRT5-negative/KRT17-positive epithelial populations for the corresponding human observations. Neither a shared stress signature nor an inferred trajectory establishes irreversible senescence or a common susceptibility to clearance.

Functional perturbations show that some abnormal epithelial states can recover without cell removal. IRE1α inhibition reduced damage-associated progenitors and increased lineage-labeled AT1 differentiation in bleomycin-injured mice [37]. P21 knockdown also restored AT2 renewal and differentiation after repeated injury [38]. Conversely, adult AT2-specific Sin3a deletion induced senescence and progressive fibrosis, which was attenuated by disrupting p53 signaling [39]. These results support comparing recovery of progenitor function with selective killing, rather than assuming that every stress-associated intermediate should be eliminated.

Human culture findings likewise indicate that progenitor function should be assessed alongside the surrounding cells. IPF-derived AT2 colony formation declined with disease stage [40], but another study found impaired organoid growth in mixed IPF suspensions rather than isolated epithelial fractions [41]. This difference cautions against attributing a culture defect exclusively to the epithelial compartment. In a separate model, HPS1-deficient respiratory-airway progenitors derived from pluripotent cells showed abnormal differentiation and activated co-cultured mesenchyme [42]. Conversely, expandable basal cells persisted in selected small bronchi of IPF lungs, although transplantation observations in three patients were exploratory [43]. The studies therefore describe impaired growth and epithelial–mesenchymal effects alongside residual expandable progenitors. Assessing the abundance and function of these residual populations before depletion can help identify cells that should instead be preserved or supported.

### 3.4. Macrophage Recruitment and Function

Macrophage selection also depends on function and disease stage because these cells can sustain fibrosis, remove damaged material or support repair. Single-cell profiling and functional experiments identified transitional CX3CR1-positive/SiglecF-positive populations [44]. Time-course studies showed changes in macrophage populations and metabolic programs [45], while a separate bleomycin study found enriched fibrotic signaling in monocyte-derived interstitial macrophages [46]. These observations make cell state and origin relevant to targeting, but activation markers do not necessarily identify disease drivers. For example, ApoE marked activated myeloid cells in a surfactant–protein-C–mutant model, yet its deletion had limited effects on disease [47]. Profiles and predicted communication can therefore identify candidate processes, whereas perturbation is needed to determine whether a given process contributes to fibrosis.

Distinguishing metabolic activation, matrix injury and recruitment helps define which macrophage process an intervention should modify. S100A8/S100A9-positive populations were associated with fibrogenic and glutamine-related programs [48]. Iron handling provided another link to fibroblast state. SLC40A1- and HAMP-expressing macrophage populations were identified, and iron plus HAMP altered transition markers in IPF-derived fibroblast cultures [49]. Tissue injury and population expansion were examined more directly. LGMN-positive macrophages degraded basement-membrane collagen IV and promoted fibroblast activation, while genetic or pharmacological interference reduced mouse fibrosis [50]. In a continuous-bleomycin model, neutrophil depletion or CSF1 neutralization reduced expansion of newly recruited monocyte-derived alveolar macrophages [51]. Diverse human T-cell populations near abnormal epithelium further placed macrophage targets within a broader immune environment [52]. The findings support targeting a defined macrophage process rather than treating all macrophages as a single fibrogenic population.

Table 1 summarizes the disease-associated features of these cell populations and the functions that selective treatment should preserve.

## 4. Senescence and Resistance to Cell Death

### 4.1. Identifying Senescent Cells

Senescence-associated targets require complementary measurements because p16, p21, DNA damage and secretory activity capture different responses. p16 and p21 report cell-cycle regulation, DNA-damage markers report lesions or damage responses, and secretory changes can also accompany inflammation; none alone demonstrates a uniform senescent state. MICSE guidance therefore recommends combinations interpreted by cell type and tissue [12]. AT2 telomere shortening and p16/p21 expression occurred in both IPF and non-IPF usual interstitial pneumonia [56]. After irradiation, IPF fibroblasts accumulated more DNA-damage lesions than healthy-donor cells but mounted weaker p53–p21 and selected secretory responses [57]. Target assessment should combine proliferation arrest, persistence, lysosomal or damage measures and functional secretion, and should retain the term senescence-like when functional validation is absent.

This variability spans processes involved in DNA maintenance, signaling and mitochondrial function. Reduced DNA-PKcs activity accompanied senescence in cultured IPF mesenchymal-progenitor-derived fibroblasts [58], whereas FBW7-dependent TPP1 degradation promoted telomere damage and epithelial dysfunction in experimental systems [59]. Other studies linked epithelial signaling to responses in surrounding cells. PAI-1 activated a p53–p21–Rb program in AT2 cells [60], and AT2 IGF-1 receptor deletion in a radiation-injury model reduced epithelial senescence and alternative macrophage activation; the human specimens in that study came from treated lung-cancer patients [61]. IGFBP2 loss also impaired alveolar function [62]. Mitochondrial studies extended these observations to ANT1 deficiency, which altered senescence in epithelial cultures and worsened fibrosis in global-knockout mice [63], and SIRT5 overexpression, which reduced mitochondrial dysfunction and senescence-associated measures in experimental alveolar cells [64]. Although these findings identify several processes amenable to experimental intervention, their interpretation depends on the cell type and model used.

Senescence-directed interventions consequently need to be classified by their observed action. Cell-directed mTOR activation produced different combinations of fibrosis and emphysema, whereas senolytic treatment had modest structural effects in naturally aged mice [65]. Fibroblast NUDT21 depletion altered alternative polyadenylation, STAT3 signaling and secretion but produced a partial or noncanonical senescence phenotype [66]. These responses should not all be described as senescent-cell clearance. A reduction in secretory activity supports functional modulation; loss of marker expression leaves cell fate unresolved without independent death or persistence measurements; and elimination requires independent evidence of death or physical removal. For immune-mediated clearance, effector dependence must additionally be established.

### 4.2. Paracrine Signaling and Apoptosis Resistance

The paracrine effects of senescence depend on the secreted product and recipient cell population, rather than uniformly promoting fibrosis. Leukotrienes from senescent fibroblasts increased collagen expression in recipient fibroblast cultures, whereas later prostaglandin production opposed some fibrogenic responses [67]. Extracellular vesicles provided another connection between stromal injury and epithelial dysfunction. IPF-fibroblast vesicles transferred microRNAs associated with SIRT3 suppression, mitochondrial injury and senescence in airway epithelial cultures [68]. Vesicles from diseased bronchial epithelium also impaired barrier measurements and induced senescence-associated changes, although the effects of individual microRNAs remained unresolved [69]. Senescent human fibroblasts and their secretome further initiated progressive fibrosis in immunodeficient mice [70], demonstrating paracrine activity under impaired adaptive surveillance. Thus, both the direction of the response and the identity of the recipient cell matter when selecting secretory activity for suppression.

Because several cell populations can supply secreted signals, targeting one source may not remove all relevant activity. Recombinant granzyme K and conditioned media from irradiated CD8 cells induced senescence-associated changes in mouse epithelial and macrophage lines. PPACK treatment at weeks 12–16 after thoracic irradiation reduced fibrosis, although the inhibitor was not GZMK-specific and genetic validation was absent [71]. These findings implicate an immune-cell-associated route, but the specific contribution of GZMK in vivo remains unresolved. Epithelial HE4–annexin-II signaling and macrophage vesicle-mediated Wnt10b suppression implicated additional routes linking injury to stromal activation or impaired renewal [72,73]. Eliminating one secretory source may reduce its contribution; sustained benefit also depends on whether other cells continue to supply the relevant signals.

Recognition and susceptibility to death are separate requirements for immune clearance. Senescent IPF fibroblasts resisted Fas ligand and TRAIL; quercetin increased ligand-induced apoptosis in culture but had little killing effect alone [74]. In normal primary fibroblasts, IPF-derived anti-HSP70 antibodies increased Bcl-2 expression and resistance to staurosporine through MOF-associated histone regulation [75]. Stiff-matrix MDM4 signaling also suppressed p53-dependent apoptosis and macrophage-mediated removal [76]. These experiments identify potential resistance mechanisms. By contrast, LAMP1-directed dual antibody–drug conjugates demonstrated target-directed killing in culture [77], but their drug-mediated cytotoxicity is not evidence of immune-effector-mediated elimination. The findings support matching a recognition strategy to measured death susceptibility, while keeping pharmacological killing distinct from immune clearance.

## 5. Antigen Recognition and Target Selection

Antigen selection must address both which cells can be recognized and which should be removed. An accessible antigen must be enriched in cells that sustain disease and limited in populations needed for repair. Because the same marker can occur in cells with different roles, target selection should consider cell identity, tissue location and function together [11,12].

### 5.1. Peptide Presentation and Antifibrotic Vaccination

Peptide vaccination recruits endogenous cytotoxic T lymphocytes (CTLs) and can recognize intracellular source proteins, but pulmonary efficacy must be interpreted against the time at which immunity is established. Major histocompatibility complex (MHC) class I molecules present intracellular peptides to CTLs. Sobecki and colleagues induced antifibrotic immunity with ADAM12- and GLI1-encoding vaccines, but immunization preceded bleomycin in the lung experiments. Treatment of established fibrosis and CD8-depletion experiments were performed in the liver [78]. The pulmonary evidence therefore concerns prevention of injury-associated fibrogenesis; neither delayed treatment of established lung fibrosis nor pulmonary CD8 dependence was established by that study.

Bai and colleagues used human and mouse immunopeptidomes with single-cell profiles to select MAF 116–124 (ALISNSHQL), APBB2 70–78 (YALTNIQAA) and TNS3 119–127 (SSYMHFTNV) [15]. The main mouse regimen combined vaccination on days 0, 7 and 14 with bleomycin on day 1 and therefore tested prevention or early interception. A separate MAF regimen began on day 7 after bleomycin, with further doses on days 14 and 21, and reduced hydroxyproline at day 28. This early post-injury experiment extends the evidence beyond preinjury vaccination, but does not establish efficacy against persistent fibrosis. In separate human assays, MAF-specific CTLs from healthy HLA-A*02:01-positive donors lysed HLA-matched IPF myofibroblasts and IL-4-polarized macrophages. Thus, presentation, mouse antifibrotic activity and human target-cell killing were demonstrated in different experiments.

These complementary assays establish a rationale for vaccination, not clinical efficacy or broad antigen safety. Immunopeptidomics verifies presentation; the human CTL assays verify recognition and killing in the tested HLA context; and mouse interventions measure effects on experimental injury. Before translation, peptide–HLA abundance should be compared in diseased, healthy and repairing cells. HLA restriction limits patient coverage, while expression of the same self-antigen during normal repair could expose useful cells to a vaccine-induced response. Unlike a short drug exposure, an induced immune response may outlast dosing, making target distribution during subsequent injury an important safety question.

Interpreting peptide enrichment is also important when prioritizing candidates. Enrichment may reflect expansion of the relevant cells, increased presentation within individual cells or inflammation-induced changes in antigen processing. These explanations have different implications for which cells a vaccine might eliminate outside a fibrotic lesion. Although transcriptomic enrichment can guide candidate selection, direct peptide–HLA measurements are needed to establish selectivity. Comparison with normal repair can then relate antigen presentation to the functions that treatment should preserve. The strongest basis for vaccine-target selection therefore combines disease-associated presentation with assessment of antigen distribution during repair.

### 5.2. Surface Antigens and FAP Function

Human FAP measurements primarily establish target distribution, not the benefit of removing FAP-expressing cells. Histological studies localized FAP to IPF remodeling sites and collagen-producing fibroblasts across interstitial lung diseases [79,80]. Exploratory FAPI-PET enrolled four transplant candidates with advanced ILD; the uptake–staining analysis involved 29 specimens from three of those patients [81]. The specimens are repeated tissue samples, not 29 independent patients, and the findings do not establish diagnostic performance or therapeutic response in IPF. In a 149-patient IPF cohort, serum FAP was not associated with severity, progression or survival when analyzed as a continuous variable [82]. Tissue accessibility and the function of FAP-bearing cells therefore require direct assessment rather than inference from circulating FAP or tracer uptake alone.

FAP protein loss and depletion of FAP-expressing cells test different mechanisms. FAP-deficient mice accumulated collagen fragments and developed worse bleomycin- and irradiation-associated fibrosis; biochemical experiments implicated FAP processing in collagen degradation and uptake [83]. This supports a matrix-clearance function of the protein, without demonstrating that every FAP-positive cell is reparative. Kimura and colleagues instead depleted FAP-positive cells with retrovirally engineered FAP-CAR T cells. Treatment began two weeks after six bleomycin doses, followed by a second infusion two weeks later; collagen increased in this established repetitive-injury model. The same targeting approach had largely unchanged histology but a modest soluble-collagen increase in an adenoviral TGF-β model [84]. Together, the experiments show that both the molecular function removed and the injury model affect the net response to FAP targeting.

**Figure 2:**
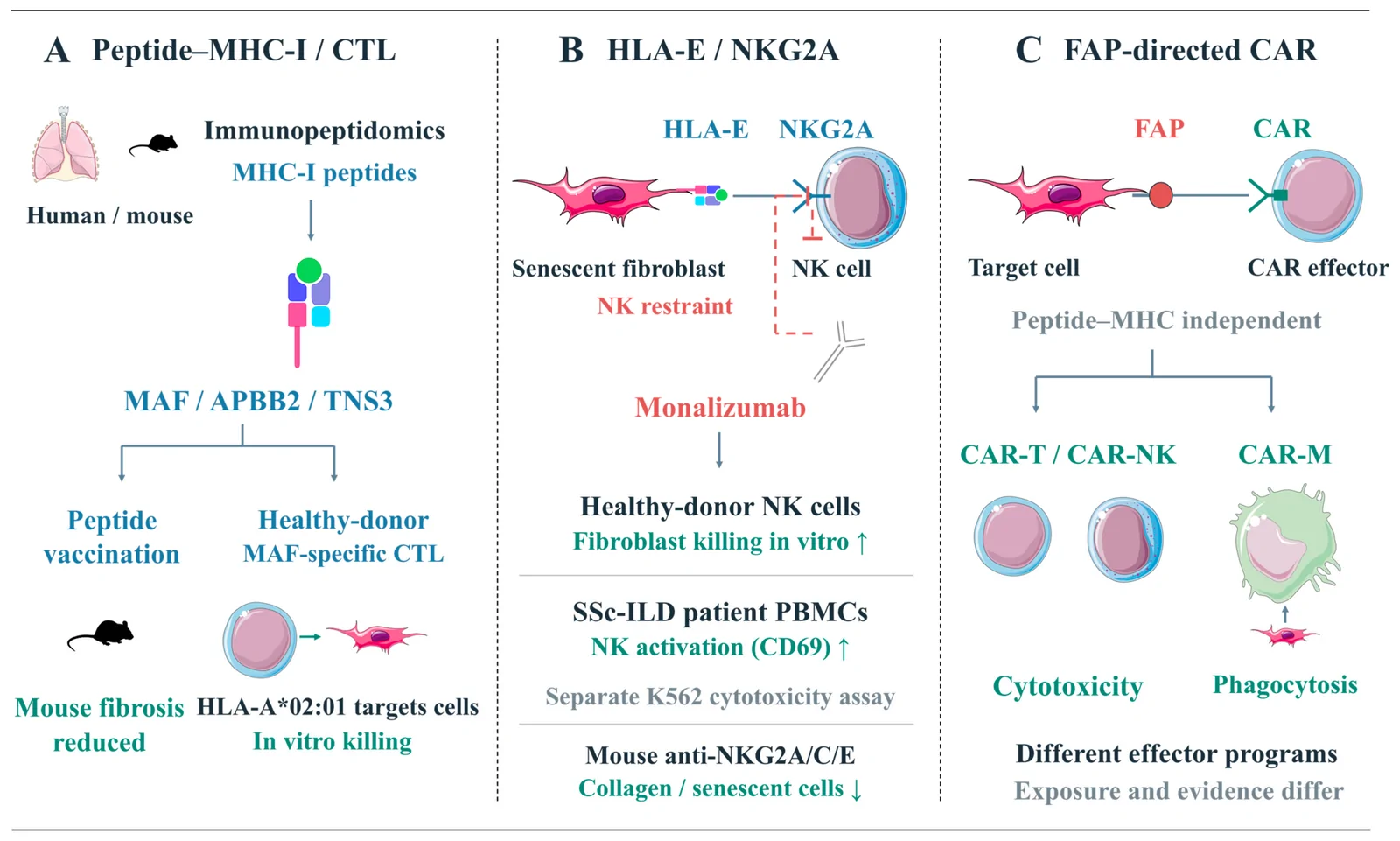
Antigen recognition and inhibitory checkpoints in pulmonary fibrosis. (**A**) Immunopeptidomics identifies fibrosis-associated MHC-I peptides. Vaccination with MAF 116–124 (ALISNSHQL), APBB2 70–78 (YALTNIQAA) or TNS3 119–127 (SSYMHFTNV) reduced experimental fibrosis in mice. In a separate human assay, MAF-specific CTLs generated from healthy HLA-A*02:01-positive donors killed HLA-matched IPF myofibroblasts and IL-4-polarized macrophages. (**B**) HLA-E on senescent fibroblasts can inhibit NKG2A-positive NK cells. Monalizumab enhanced fibroblast killing by healthy-donor NK cells. SSc-ILD patient-derived peripheral blood mononuclear cells (PBMCs) showed enhanced NK activation during fibroblast coculture; a separate cytotoxicity assay used K562 targets. These patient assays did not assess killing of autologous IPF fibroblasts. Mouse anti-NKG2A/C/E treatment reduced collagen and senescent-cell abundance in a separate lung-injury experiment. Green upward (↑) and downward (↓) arrows indicate increases and decreases, respectively, in the labeled readouts relative to the corresponding experimental controls. (**C**) FAP-directed CARs recognize surface antigen without peptide–MHC matching. CAR-T, CAR-NK and CAR macrophages differ in cytotoxic or engulfment activity, duration of receptor expression and pulmonary evidence [10,13,15,16,89,90,91]. Selected cell, lung and antibody icons were adapted from Servier Medical Art (CC BY 3.0), and MHC-I symbols from Helicase 11 (CC BY 4.0), via Bioicons. Mouse icons by Ben-Murrell were obtained via Bioicons (CC0 1.0). Icons were resized and assembled for this schematic; the original domain labels on the MHC-I symbol were removed and its orientation adjusted.

Alternative surface targets broaden the range of recognizable cells, although evidence for target recognition remains stronger than evidence for treatment of established pulmonary fibrosis. uPAR-directed CAR-T cells cleared senescent cells in mouse liver fibrosis and lung cancer, and subsequent work extended the approach to aging and metabolic dysfunction [85,86]. A uPAR-binding peptide that recruits immune cells also reduced senescence-associated signals and collagen after early bleomycin lung injury [87]. Evidence in established pulmonary disease nevertheless remains limited. Other candidates have been tested in more restricted systems. LAMP1-directed antibody–drug conjugates were evaluated in culture, whereas GPNMB-CAR-T cells were evaluated against senescent endothelial cells and in subcutaneous Matrigel implants containing targets and effectors [77,88]. For each candidate, the disease context and experimental system determine what evidence of recognition contributes to pulmonary treatment development. For each candidate, the disease context and experimental system determine what evidence of recognition contributes to pulmonary treatment development. Figure 2 summarizes the recognition mechanisms and supporting evidence for peptide vaccination, NKG2A blockade and FAP-directed CAR targeting.

## 6. Immune Checkpoints in Fibrotic Lung Tissue

An immune encounter can favor target-cell clearance or effector-cell loss, depending on signaling direction and injury phase. Fibrotic lung myofibroblasts illustrate the latter outcome. They resisted Fas-mediated apoptosis and used Fas ligand (FasL) to kill Fas-positive lymphocytes [92]. Conversely, immune-cell FasL induced myofibroblast apoptosis and supported resolution in coculture and mouse reconstitution experiments [93]. Proximity between immune cells and myofibroblasts permits contact, but does not determine which population will be eliminated. Ligand expression, the identity of the cell receiving the signal and the phase of injury are therefore needed to interpret this pathway.

### 6.1. HLA-E/NKG2A and NK-Cell Suppression

Reduced NK surveillance can involve inhibitory receptor signaling and soluble influences from diseased tissue. In culture, senescent dermal fibroblasts expressed HLA-E and inhibited NKG2A-bearing NK cells and differentiated CD8 T cells. Receptor blockade restored responses in that setting [94]. However, the local IPF environment also affected effector function. Conditioned medium from fibroblasts in end-stage lower lobes impaired NK cytotoxicity and migration, while tissue NK cells had an altered CD57/NKG2A phenotype [95]. Both studies describe impaired effector function, but they examine different sources of suppression. The findings implicate receptor engagement and soluble conditions without establishing that the same mechanism operates in the culture and lung settings.

The HLA-E/NKG2A evidence comprises distinct human and mouse observations. In human IPF, HLA-E-positive, senescence-associated HAS1 fibroblasts were enriched at the periphery of fibroblastic foci, adjacent to NKG2A-positive NK cells; core myofibroblasts lacked the same HLA-E pattern [10]. This is expression and spatial-association evidence, not a direct measurement of killing. In a separate bleomycin experiment, anti-NKG2A/C/E given on days 14, 17 and 20 reduced collagen and senescent-fibroblast abundance at day 24 without increasing measured BAL inflammation. The delayed schedule supports activity during experimental fibrogenesis, but does not establish reversal of persistent disease. Spontaneous resolution in the model and the murine antibody’s broader receptor specificity also prevent equivalence with human NKG2A blockade.

The human functional assays likewise differ by effector and target source. Monalizumab enhanced perforin-dependent killing of irradiated senescent lung fibroblasts by healthy-donor NK cells. PBMCs from patients with SSc-ILD, however, were assessed for NK-cell CD69 activation in fibroblast coculture, with cytotoxicity tested separately against K562 cells [10]. Neither patient NK activation nor K562 killing demonstrates elimination of autologous fibrotic lung fibroblasts.

Target distribution also affects what successful blockade might achieve. Bulk HLA-E expression cannot resolve the peripheral HAS1 fibroblasts from matrix-producing cells in the lesion core. NKG2A blockade could restore responses where responsive NK cells contact HLA-E-bearing targets, but neither spatial adjacency nor receptor expression establishes physical access, elimination of central myofibroblasts or preservation of nearby reparative cells. These are unresolved treatment questions. Functional experiments should therefore retain the regional cell identities and measure effector access and target-cell fate before a peripheral checkpoint response is generalized to the whole fibrotic focus.

### 6.2. CTLA4 and PD-1/PD-L1

CTLA4 blockade links human localization to separate mouse intervention and ex vivo cytotoxicity evidence. CTLA4-positive CD8 T cells were found near fibrogenic regions in human IPF. In human-CTLA4-knockin mice given two bleomycin doses 14 days apart, ipilimumab reduced collagen and senescence-associated cells, increased AT2 abundance and proliferation, and enhanced CD8-cell killing ex vivo [13]. The Results and figure legends place treatment 21 days after the second bleomycin dose, or approximately day 35 after the first, whereas the Methods state only ‘on day 21’ without specifying the reference dose. The study supports late-phase activity in a repetitive-injury model, but the precise dosing timeline cannot be resolved from these inconsistent descriptions. The ex vivo assay supports cytolytic capacity; the tissue changes do not establish that CD8-mediated clearance alone caused repair, because other CTLA4-positive populations were not excluded.

PD-L1 experiments further separate direct recognition from a model-specific antifibrotic response. In IPF mesenchymal progenitors, IL-8/CXCR2 signaling induced DNA damage, senescence and PD-L1; PD-L1 knockdown or antibody blockade increased susceptibility to NK92-cell killing in vitro. Combined NK92 transfer and anti-PD-L1 reduced fibrosis in bleomycin-primed, irradiated xenograft mice [96]. The culture assay demonstrates increased killing by an NK cell line, whereas the combined intervention does not isolate endogenous immune clearance or the effect of antibody alone. In silica-exposed mice, a PD-1/PD-L1 inhibitor improved fibrotic outcomes while the tested CTLA4 inhibitor was less effective [97]. These disease and treatment settings are complementary, but their outcomes cannot be interpreted as equivalent checkpoint effects in established IPF.

Checkpoint inhibition can also suppress profibrotic communication without demonstrating selective cell elimination. After bleomycin in MuHV-4-infected mice, PD-1-positive CD8 T cells accumulated near PD-L1-positive fibroblasts; anti-PD-1 started three weeks after injury and continued for four weeks, reducing collagen [98]. In separate human cocultures, activated CD8 T cells from EBV-seropositive donors increased profibrotic gene expression in MRC-5 fibroblasts, which anti-PD-L1 prevented. The measured human response is functional modulation of a fibroblast–T-cell interaction, not target-cell death. Likewise, the delayed mouse collagen response supports an antifibrotic effect in a virus-associated injury model without establishing selective senescent-cell clearance or efficacy in persistent human IPF.

Experimental benefits must be considered alongside pulmonary immune risks. Restoring surveillance and inducing generalized activation can have different consequences. Cancer-associated checkpoint pneumonitis has several radiological presentations, including organizing pneumonia, and variable clinical courses [99,100]. Separate studies also identified CD155-dependent cytotoxic activity in a CD57-positive CD8 subset from SSc-ILD and virus-associated CD8–macrophage signaling that sustained an abnormal epithelial progenitor population [14,53]. Immune activity can thus remove abnormal cells or injure tissue. Its cellular consequences and effects on lung function must both be assessed in checkpoint-directed treatment.

### 6.3. Phagocytosis and Nonprotein Inhibitory Signals

Macrophage uptake assays require a defined substrate before they can support a claim of cell clearance. CD47 staining in remodeled UIP epithelium, including p16-positive cells, identifies a candidate inhibitory signal but does not measure resistance to engulfment [101]. In silica-exposed mice, RRx-001 reduced fibrosis-associated outcomes and altered macrophage polarization. The functional assay measured FITC-dextran uptake, a general endocytic readout, rather than phagocytosis of identified senescent target cells [102]. We therefore classify this evidence as modulation of uptake and macrophage activity.

Nonprotein ligands can suppress immune function without changing effector abundance. Senescent-cell GD3 inhibited NK degranulation, and anti-GD3 begun on day 5 after bleomycin restored intrapulmonary NK activity while reducing senescence-associated signals and collagen at day 27 [103]. Paired micro-CT at days 14 and 27 followed the lungs during treatment; improved imaging and marker counts did not directly trace the elimination of individual targets. In contrast, antibodies against isoDGR-containing damaged proteins targeted extracellular damage and reduced lung inflammation [104]. The first approach modulates a cell-associated inhibitory signal, whereas the second addresses damaged extracellular material; neither should be described simply as removal of a defined senescent lung-cell population.

## 7. Immune-Directed Treatments

### 7.1. Restoring Endogenous Immune Function

Human NK-cell associations vary by compartment and cannot determine whether increasing NK abundance is therapeutic. One IPF cohort had reduced circulating NK cells, whereas BAL samples showed higher NK proportions in IPF than in other fibrotic ILDs [105,106]. Longitudinal blood transcriptomics associated inferred NK-cell changes with subsequent FVC decline [107]. These are different measurements in different patient groups, rather than opposing intervention results. They support assessing tissue location, per-cell function and the target population before deciding whether an NK-directed treatment should increase cell numbers, release inhibition or alter secretory activity.

The mouse depletion studies also differ in perturbation and treatment phase. Anti-NK1.1 beginning on day 14 after intratracheal bleomycin increased collagen and senescence-associated populations at week 7 [95]. Anti-asialo-GM1 did not alter fibrosis when given before and throughout injury or during days 10–21, and the Ly49A-transgenic NK-deficient model likewise showed no material fibrosis change after osmotic-minipump bleomycin or irradiation [108,109]. NK1.1 and asialo-GM1 are not exclusive functional-subset markers, so depletion specificity and residual populations must be considered alongside strain, injury delivery and endpoint. The experiments have not isolated these variables in matched comparisons. Human coculture methods that quantify per-cell killing [110] could complement subset-resolved depletion and cytokine measurements, distinguishing loss of cytotoxic surveillance from altered soluble signaling.

Releasing an inhibitory checkpoint and increasing an activating signal are not equivalent tests of NK function. Human MICA polymorphisms and reduced BAL NKG2D expression were associated with IPF [111], while NKG2D/perforin-dependent killing was demonstrated in senescent renal epithelial cultures [112]. In a lung model, however, NKG2D overexpression worsened fibrosis and blockade improved outcomes; whole-lung p53/p21 measurements did not resolve the responding cell population [113]. This does not negate the NKG2A findings [10]. NKG2A blockade tests disinhibition of effectors near HLA-E-bearing targets, whereas NKG2D manipulation can alter responses to a different ligand set. The net pulmonary result depends on which cells receive the signal and whether cytotoxic or cytokine-mediated activity predominates; these mechanisms require cell-specific tests rather than inference from receptor abundance.

Effector restoration can target a demonstrated clearance defect, although its tissue consequences still need separate attribution. Excessive Cdc42 activity in aged human NK cells impaired granule release, and pharmacological rebalancing improved senescent-fibroblast killing in culture [114]. In macrophages, loss of chaperone-mediated autophagy (CMA) impaired SIRPα internalization and lysosomal degradation, increased inhibitory signaling and reduced phagocytosis and efferocytosis; fibroblast secretomes further suppressed these functions [115]. Systemic CA77.1 from day 2 after bleomycin reduced fibrosis at day 15, whereas treatment from day 7 was less effective. These are early post-injury schedules, not confirmed reversal of established pulmonary fibrosis. The cellular experiments support a clearance mechanism, but the systemic lung response cannot be attributed exclusively to macrophage removal of senescent cells.

Soluble immune activity can also restrain fibrosis without greater cytotoxicity. Invariant natural killer T (iNKT) cells are T lymphocytes and should not be conflated with conventional NK cells. Early iNKT studies linked protection to IFN-γ-dependent suppression of TGF-β [116]. α-Galactosylceramide given on day 10 after bleomycin reduced senescence-associated epithelial and mesenchymal cells, collagen and mortality, with lung-cell assessment at day 14, but direct killing was not fully separated from cytokine effects [117]. Conventional NK cells treated with IL-12 likewise reduced fibroblast collagen and α-SMA through soluble factors, including IFN-γ, despite lower cytotoxicity [118]. These findings support functional immune modulation as a potentially useful antifibrotic outcome, distinct from proven immune-mediated elimination.

### 7.2. FAP-Directed CAR-T Cells and Cytolytic Tregs

FAP-directed T-cell approaches have shown antifibrotic activity with different delivery strategies and treatment settings. Mouse cardiac studies provided complementary findings. FAP-CAR-T cells reduced fibrosis and improved function after cardiac injury, and CD5-targeted lipid nanoparticles subsequently delivered CAR mRNA to T cells in vivo, producing transient receptor expression [119,120]. These studies established a method for stromal targeting and a rationale for examining exposure duration. Pulmonary effects still required separate assessment, because activity in an injured heart does not establish how a fibrotic lung will respond.

The transient FAP-CAR-T lung study combined matrix and epithelial responses in an early fibrotic phase. CD5-targeted CAR mRNA nanoparticles given on days 7 and 14 after bleomycin in young and aged mice reduced FAP-positive fibroblasts, collagen and lung stiffness and changed epithelial profiles at day 28 [16]. Group-level histology at day 7 already showed fibrosis and alveolar abnormalities, supporting treatment after fibrosis had begun. Fewer transitional states and more proliferating progenitor-like cells accompanied the stromal response, but marker-positive counts do not independently resolve the fate of each targeted cell. The inferred epithelial trajectories are not lineage tracing, and transient receptor expression does not establish preservation of normal FAP-positive cells.

The contrasting FAP studies therefore do not identify exposure duration as the explanation for benefit or harm. FAP-CAR depletion worsened collagen accumulation in established repetitive-bleomycin fibrosis [84], whereas transient CAR mRNA was tested on days 7 and 14 after a different injury regimen [16]. The studies differed in construct, effector preparation, disease phase and affected populations, as well as receptor duration. Loss of FAP-dependent collagen processing is supported by deficiency experiments [83]; differential preservation of reparative stroma is a plausible additional explanation, not a mechanism demonstrated by comparing these treatment studies. Matched experiments must distinguish these possibilities. Safety also extends beyond the lung because FAP-reactive CAR-T cells recognized marrow stromal cells and caused cachexia and bone toxicity in mice [121].

CAR-cTregs combine target-directed cytolysis with immune suppression. FAP-directed, FoxP3-engineered cells killed activated fibroblasts through granzyme/perforin-associated mechanisms and suppressed conventional T-cell responses. Day-14 infusion reduced bleomycin fibrosis, whereas day-1 infusion did not improve key outcomes and increased some collagen measurements [91]. The study reported that CAR-cTreg infusion on day 28 reduced lung hydroxyproline content and histological fibrosis at day 44; separate pretreatment fibrosis quantification was not shown for that experiment. These findings support a treatment-phase-dependent response, including later post-injury activity. The authors reported less inflammation than with cytotoxic CAR-T comparators and persistence for at least one month. Persistent effector activity warrants assessment of phenotype stability, normal wound healing and late toxicity; a human cytokine-release safety advantage has not been established.

### 7.3. CAR-NK Cells and CAR Macrophages

CAR-NK evidence is strongest for antigen-dependent cytotoxicity in culture, with much more limited pulmonary intervention evidence. Primary human FAP-CAR-NK cells acted against activated fibroblasts and in human lung-organoid coculture [90]. In mice, bleomycin was followed by 10 Gy fractionated whole-body irradiation on day 7, NK-cell infusion 72 h later and tissue assessment another 72 h after infusion. Deaths in all groups left two tissue samples per group, and there was no mock-CAR control. This acute feasibility experiment can inform lung access and target engagement, but it does not establish durable cell clearance, fibrosis reversal, normal-tissue safety or recovery of gas exchange. Nor does the short observation determine whether persistence is sufficiently controllable for chronic pulmonary disease.

CAR macrophages add engulfment and matrix handling to the effector functions under study. FAP-CAR macrophages engulfed activated fibroblasts and degraded collagen in culture. Viscoelastic hydrogel priming lowered membrane tension, redistributed CARs and enhanced signaling; primed cells infused on days 7 and 14 after bleomycin improved fibrosis-associated outcomes at day 21 [89]. The culture experiments establish these effector activities, whereas the mouse result establishes an early post-injury antifibrotic response. It does not isolate fibroblast engulfment or direct matrix degradation as the cause of that response. This contrasts engulfment with CAR-T/CAR-NK cytolysis; activity in stiff human fibrotic tissue remains untested.

Donor-cell studies show why cell composition matters alongside CAR activity when interpreting an antifibrotic response. A myeloid-CAR-knockin study used Lyz2-Cre to express FAP-CAR in monocytes, macrophages and granulocytes; donor mice were resistant to induced fibrosis. Transfer of donor macrophages or whole blood on day 6 after bleomycin reduced fibrosis in recipients assessed on day 21 [122]. Both approaches produced an early post-injury effect in mice, but whole blood contained several CAR-positive myeloid populations, so its effect cannot be assigned exclusively to macrophages. The experiments support early transfer-based intervention rather than treatment of established human fibrosis, and interpretation of benefit depends on which cells were transferred.

### 7.4. Comparing Treatment Effects

Across strategies, the main trade-off is between the breadth and duration of immune recognition and the functions that remain after treatment. Vaccines exploit measured peptide presentation but depend on patient HLA and endogenous CTLs. Checkpoint antibodies require accessible targets and responsive endogenous effectors; their benefit may reflect restored killing or altered signaling. CARs redirect selected effectors toward surface antigen without peptide–HLA matching, but transfer the problem of selectivity to antigen distribution, effector activity and persistence. Macrophage-directed approaches can additionally alter uptake and matrix handling. Because resolving fibroblasts can support epithelium [9], no platform should be prioritized by target-cell loss or collagen reduction alone. The most informative comparison tests target fate, preservation of reparative populations and lung function under the same disease conditions.

Exposure cannot yet be used to rank these platforms clinically. Vaccine-induced immunity may outlast dosing, checkpoint inhibition depends on the endogenous immune compartment, and transferred or stably engineered cells can retain activity after administration. Transient CAR mRNA limits receptor expression, but effector survival and normal wound-healing safety require separate assessment. The transient FAP-CAR-T, persistent CAR-cTreg, acute CAR-NK and early CAR-macrophage studies differ too greatly in model and follow-up to identify an optimal duration. Future comparisons should vary exposure within a fixed target, construct, effector program and established-disease model, then assess recovery and off-tissue injury after treatment withdrawal.

Table 2 compares the main pulmonary interventions by target, evidence source, treatment timing and measured outcome.

## 8. Matrix Remodeling and Alveolar Repair

### 8.1. Collagen Turnover and Tissue Mechanics

The opposing FAP results show why removal of a stromal population and recovery of its matrix cannot be assumed to coincide. FAP processed metalloproteinase-generated collagen fragments and promoted uptake; constitutive FAP deficiency impaired this function and worsened bleomycin- and irradiation-associated fibrosis [83]. By contrast, FAP-directed CAR-T depletion removed antigen-bearing cells and aggravated established repetitive-BLM fibrosis, with few histological changes in an adenoviral TGF-β model [84]. Transient FAP-CAR-T treatment produced antifibrotic responses in a different single-injury setting [16]. Enzymatic collagen processing is therefore a demonstrated explanation for the FAP-loss phenotype, whereas preservation of reparative stroma, exposure duration and target composition remain plausible explanations for differences among cell-targeting studies. These hypotheses require matched experiments rather than inference from the direction of collagen change alone.

Matrix remodeling also changes the cellular context in which clearance occurs. LGMN-positive macrophages degraded basement-membrane collagen IV and promoted fibroblast activation and collagen-I deposition [50]. Conversely, vitronectin altered macrophage metabolism and fibrogenic activity in three-dimensional culture, with human localization and knockout-mouse observations providing complementary evidence [124]. Matrix-directed modulation may thus be useful when the remaining cells retain reparative potential. Soft substrates and spheroids shifted IPF-derived fibroblasts toward a reversible lipid-containing state [125], whereas the related culture effects of desipramine did not translate consistently across more complex systems [126]. These findings favor measuring both the residual matrix and the functions of surviving cells when comparing depletion with reprogramming.

Residual stiffness can sustain dysfunction even after the abundance of a candidate target falls. Stiffer alginate increased nuclear YAP and matrix-associated transcripts in MRC-5 fibroblasts [127]. In primary human AT2 cells, stiffness was linked to DNA damage and nuclear changes [128]. In separate mouse AT2 cultures, high stiffness promoted senescence through lysosomal degradation of lamin A/C, with reduced lamin A/C also observed in human IPF tissue [129]. Fibrotic rat scaffolds induced integrin-β1/FAK/YAP-associated senescence-like responses in A549 cells [130]. The latter is a carcinoma-cell model, not independent validation in primary human AT2 cells. Across these distinct systems, matrix mechanics can act on both stroma and epithelium. Recovery assessment should therefore include collagen organization, turnover and stiffness, alongside epithelial differentiation.

### 8.2. Signals Controlling Alveolar Differentiation

AT2 proliferation, acquisition of AT1-associated markers and completed differentiation are separate observations. Mouse Hippo/YAP studies used genetic lineage labels, but individual Yap and Taz perturbations produced different outcomes across experimental settings [131,132]. Sustained YAP/TAZ activation promoted persistent transitional and airway-like states [133], making cell identity, perturbation and duration more informative than a uniformly beneficial or harmful pathway label. In the GLS1 study, AT2-specific deletion and lineage tracing directly assessed AT2-derived AT1 differentiation; excess autophagy reduced YAP/TAZ, and autophagy inhibition restored differentiation and improved measured lung function [134]. These experiments provide mouse cell-of-origin evidence; they do not establish reconstruction of chronically fibrotic human alveoli.

Epithelial responses to Wnt and ROCK manipulation likewise depend on cell type and experimental setting. CasRx-mediated Axin1/Axin2 degradation activated epithelial Wnt signaling, increased AT2 proliferation and improved epithelial and fibrosis measurements after bleomycin [135]. That study tracked AAV-transduced epithelium with a Cre reporter, whereas the AT2-specific genetic designs above more directly resolve AT2-derived progeny. Fibroblast Wnt11, in contrast, supported myofibroblast differentiation [136]. In human non-IPF donor AT2 organoids, ROCK activity opposed TGF-β-induced KRT17 expression and loss of SFTPC, while IPF-tissue staining supplied anatomical support [137]. This organoid result demonstrates preservation of an epithelial state, not completed regeneration or treatment efficacy in IPF patients.

Restored alveolar function also requires a working vascular and lymphatic interface. Mouse vascular perturbations linked endothelial Jagged1-associated signaling to fibrosis and protective angiocrine signals to alveolar recovery [54]. Endothelial TPI1 dopaminylation limited ferroptosis and favored regeneration in pneumonectomy and bleomycin models [138]. Lymphatic vessels illustrated a different functional requirement: expansion coexisted with impaired tracer clearance, and VEGFR3 inhibition during weeks 4–12 after bleomycin hindered regression [55]. Thus, epithelial marker recovery should be related to intact barrier function, perfusion and drainage. Increased vascular or lymphatic density alone does not supply these measurements.

### 8.3. Cell Depletion or Functional Reprogramming

Functional reprogramming is an alternative to depletion when a population retains useful activity. BCL9 inhibition promoted fibroblast lipogenesis through macrophage–fibroblast interactions [139], targeted RNA altered fibrogenic macrophage programs [140], and AT2 transplantation changed macrophage activation in rats with established bleomycin fibrosis [141]. These interventions modify communication or cell state rather than demonstrate selective immune elimination. Fn14 further shows why the cellular context matters: whole-body loss worsened mouse fibrosis and reduced macrophage-supported AT2 proliferation [142], whereas a separate study linked Fn14 to fibroblast senescence and fibrosis [143]. The relevant comparison is therefore which cellular function is retained or suppressed by reprogramming versus removal.

The distinction between structural response and functional recovery is illustrated by senolytic experiments. Early genetic or pharmacological removal of senescent cells improved mouse pulmonary and physical function without visibly reversing fibrosis [144], whereas navitoclax reduced persistent radiation-associated fibrosis in a different model [145]. Single and repetitive bleomycin also differed in persistence, function and senescence profiles [146]. We use partial repair for improvements in individual components and reserve functional alveolar regeneration for concordant structural recovery, evidence of AT2-to-AT1 differentiation and sustained physiological improvement (Table 3).

## 9. Clinical Translation and Safety

### 9.1. Pulmonary Immune Toxicity

Pulmonary safety must be interpreted by intervention and population. In PANTHER-IPF, prednisone, azathioprine and N-acetylcysteine increased deaths and hospitalizations relative to placebo, leading to early termination of that arm [147]. This is direct clinical evidence against that combination, not against all immune-directed treatment and not a toxicity estimate for selective clearance. It establishes the need to evaluate candidate mechanisms against controlled outcomes in the intended patient population.

Systemic checkpoint inhibition has a different, established pulmonary risk in oncology. Meta-analyses reported PD-1-associated pneumonitis, tumor-dependent variation, greater incidence with combination treatment and a higher pooled incidence with PD-1 than PD-L1 inhibitors in non-small-cell lung cancer [148,149]. A randomized-trial synthesis found increased pneumonitis with PD-1/PD-L1 inhibitors versus chemotherapy and with nivolumab–ipilimumab versus monotherapy; the ipilimumab-only comparison was not significant [123]. These data justify pulmonary monitoring, but they do not quantify NKG2A-blockade risk in IPF. The short mouse NKG2A experiment found no increase in its measured BAL inflammatory indices [10]; this is limited direct pulmonary safety evidence, not exclusion of delayed toxicity.

For FAP-directed engineered cells, recognition of marrow stromal cells and cachexia has been demonstrated in mouse immunotherapy experiments [121]. This is direct evidence of on-target injury outside the lung, not a clinical IPF toxicity rate. Normal antigen-bearing stroma, vascular-supporting cells and cells engaged in wound healing should therefore be assessed alongside the fibrotic target. Unintended antigen recognition, cytokine-mediated lung injury and prolonged activity are additional product-specific concerns whose pulmonary magnitude remains incompletely characterized. Vaccines likewise require assessment of cross-reactivity and durable immunity to self-antigens. Transient or switchable receptor expression offers an exposure-control strategy, but preservation of repair and safety after a subsequent injury require direct testing, including after dosing ends.

### 9.2. Pharmacological Senolysis as a Clinical Comparator

Pharmacological senolysis is included as a clinical comparator for the cell-removal concept, not as clinical validation of selective immune clearance. Dasatinib plus quercetin acts through pharmacological effects on senescence-associated survival pathways; these IPF studies did not test antigen recognition or immune-effector-dependent killing. An open-label study in 14 patients reported improvement in some physical-function measures without a pulmonary-function change [150]. A randomized placebo-controlled pilot in 12 patients primarily assessed feasibility and tolerability and did not establish functional efficacy [151]. Both studies were small and exploratory, with no demonstration of defined target-cell elimination in the lung or histological reversal of IPF.

In diabetic kidney disease, dasatinib plus quercetin reduced senescence-associated measurements in sampled adipose tissue and skin [152]. These extrapulmonary findings inform pharmacological activity but cannot establish clearance of IPF lung cells or identify an immune-effector mechanism. Together with the IPF pilots, they support testing pulmonary target engagement and prespecified lung outcomes in subsequent studies. General physical-function improvement and systemic biomarkers should remain separate from evidence of alveolar repair.

### 9.3. Treatment Timing and Model Selection

Efficacy depends on the model stage at which treatment begins. Single-dose bleomycin passes through acute injury and inflammation into fibrosis and often resolution; early dosing can therefore prevent fibrogenesis without removing an established scar. Preventive designs predominated in a historical survey [153]. Repetitive injury does not by itself guarantee persistence: under the same regimen, prolonged fibrosis occurred in DBA/2 and endothelial nitric oxide synthase-deficient C57BL/6J mice but not wild-type C57BL/6J mice [154]. Complementary models differ in initiating injury, inflammation and natural history [155], so reversal of persistent fibrosis requires its presence and course to be documented before treatment.

For each candidate, the ATS recommendations support treatment after acute inflammation, biochemical as well as histological outcomes, complementary models and human validation [18]. Table 2 applies this stage distinction across strategies. Baseline fibrosis and target-cell abundance, age, sex, injury route and background treatment should be reported, with follow-up after withdrawal. These criteria explain why day-6 or day-7 CAR treatment and pre-injury vaccination address different questions from delayed treatment in a characterized persistent-fibrosis model.

Human systems contribute different information at each stage. IPF tissue can establish lesion-associated target distribution, whereas healthy-donor effectors test function under selected conditions; neither substitutes for killing of autologous targets in a patient-derived fibrotic niche. SSc-ILD assays are informative about that disease but cannot be assumed to reproduce IPF. End-stage explants also differ from earlier disease, and experimentally fibrosed donor slices differ from IPF tissue. Organoids and lung slices permit functional perturbation but incompletely reproduce perfusion, immune recruitment and mechanics. These source-specific strengths and limitations should guide the transition from human mapping to ex vivo tests and then persistent-fibrosis models.

## 10. Priorities for Further Study

Translation should begin with human target validation. The minimum evidence is reproducible localization of the candidate antigen or peptide–HLA complex in a defined disease-associated population, assessed against healthy tissue and normal repair. Cell identity, lesion position, persistence and retained epithelial-support functions should be recorded. Spatial proximity identifies potential access but does not replace functional interaction assays. A candidate should progress only when these measurements justify targeting that population and identify the normal tissues that also require testing.

The next stage is functional testing in human-cell or ex vivo systems. Antigen-dependent target recognition should be followed by observed target-cell death or engulfment, independent counts or fate measurements, and an effector-dependence control. Matched conditions should measure surviving reparative cells, inflammatory output and epithelial support. Autologous targets are preferable when feasible; healthy-donor effectors or non-IPF donor tissue answer narrower questions. If the effect is reduced secretion or state conversion without cell elimination, it should progress as a functional-modulation strategy with corresponding endpoints.

**Figure 3:**
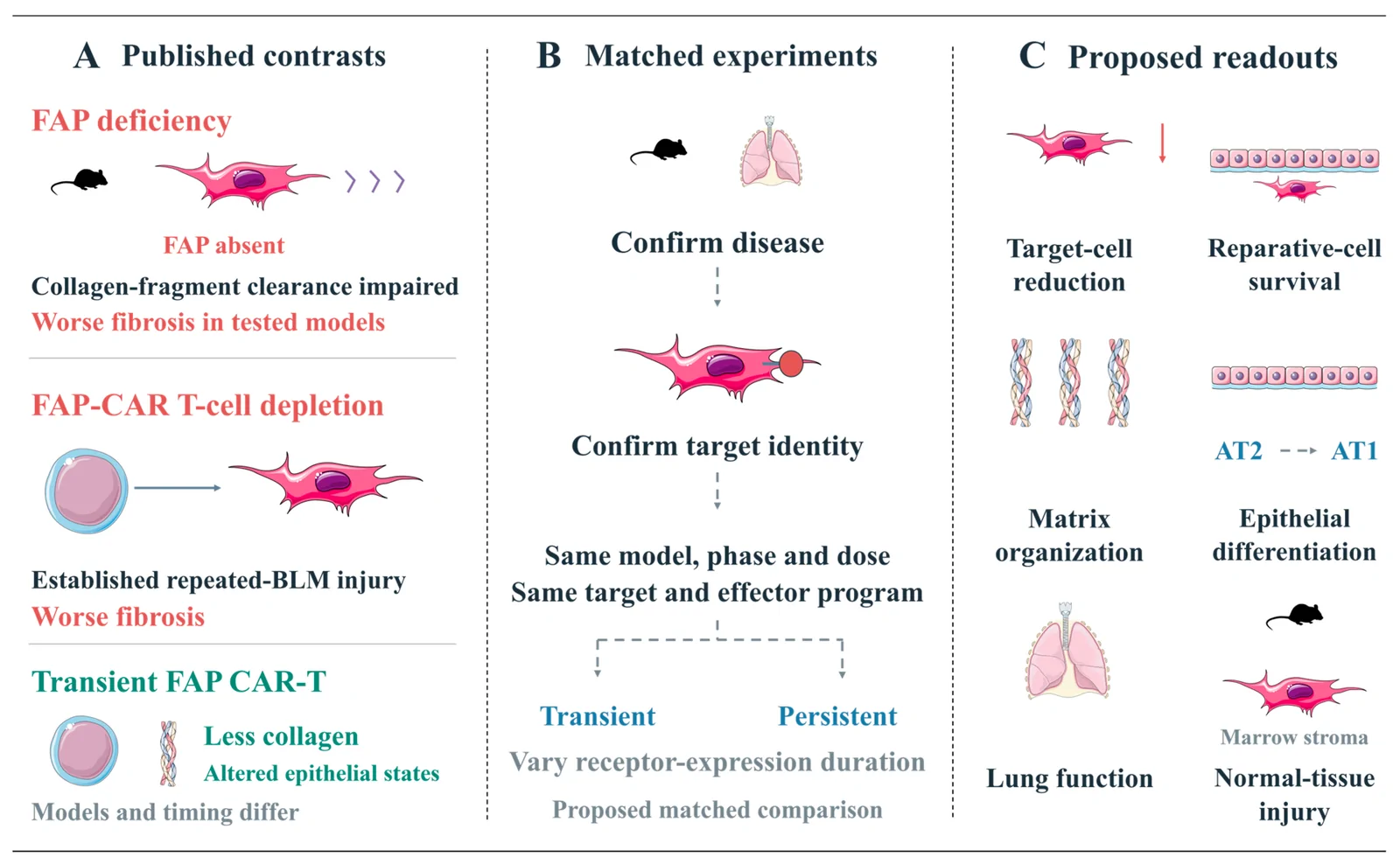
Proposed experiments to compare FAP-targeting outcomes. (**A**) FAP deficiency and FAP-directed CAR-T cell depletion increased fibrosis in some mouse settings, whereas transient FAP-CAR-T treatment improved collagen-related and epithelial-state measurements in a different model. Loss of FAP enzyme function and elimination of FAP-expressing cells are distinct interventions. The purple chevron-shaped symbols in panel A represent collagen fragments. (**B**) A comparison of exposure duration should hold the injury model, target definition, pretreatment disease stage, delivered dose and effector program constant while varying the duration of receptor expression or effector activity. Baseline fibrosis and target-cell fate should be measured. (**C**) Outcomes should assess target-cell loss, reparative-cell survival, matrix organization, epithelial differentiation and sustained lung function, together with normal-tissue injury. Dashed arrows denote proposed experiments and outcomes, not established explanations for the published differences [9,11,12,13,16,83,84,121]. Selected cell, lung and collagen icons were adapted from Servier Medical Art (CC BY 3.0) via Bioicons. Mouse icons by Ben-Murrell were obtained via Bioicons (CC0 1.0). Icons were resized and assembled for this schematic.

Persistent-fibrosis animal testing should then establish efficacy after disease and target burden are documented. The minimum assessment includes biochemical and histological fibrosis, target-cell fate, reparative-cell survival, lung physiology and normal-organ injury, with follow-up after treatment withdrawal. For exposure-duration questions, the model, target definition, disease stage, delivered dose and effector program should remain constant while receptor expression or activity duration varies. Figure 3 presents these proposed comparisons; it does not identify the cause of the opposing FAP results.

Clinical investigation would require a product-specific safety assessment, feasible exposure control and a pulmonary target-engagement plan, supported by the preceding stages. IPF and other fibrotic ILDs should be characterized separately, with prespecified background therapy, lung-function outcomes and follow-up for delayed injury. Early feasibility or biomarker signals would not alone justify a regeneration claim. Durable recovery should connect structural improvement to epithelial barrier, vascular and physiological function. Studying clearance together with regenerative treatment is reasonable only when each component and their combined effects on retained repair capacity can be evaluated.

## 11. Conclusions

Selective immune targeting has reached different points in recognition, cell elimination and tissue recovery, with the strongest pulmonary intervention evidence still experimental. The comparative evidence supports testing target function, disease stage and effector exposure together; it does not identify a strategy that reliably reconstructs chronically fibrotic human alveoli. Human target validation, functional clearance assays and treatment studies in models with documented persistent fibrosis should therefore precede clinical investigation. Sustained lung physiology and preservation of reparative populations provide the final tests of whether a clearance strategy enables useful recovery.

## Figures and Tables

**Table 1:** Disease-associated cell features and functions to preserve during selective treatment.

Cell Population	Disease-Associated Feature	Functions to Preserve	Principal Evidence and Interpretation
Activated fibroblasts	Persistent matrix production and CTHRC1-associated states	Epithelial support and resolution signals	Mouse fate mapping/transfer and experimentally fibrosed human lung slices; some stromal states are reversible or support repair [9,28].
Senescence-associated stromal cells	Persistent damage and secretory activity	Transient responses supporting airway repair	Mouse naphthalene airway-repair experiment [11]; in vivo senescence-assessment guidance [12]. Neither establishes human alveolar repair.
Transitional epithelium	Incomplete alveolar differentiation	AT2 renewal and AT1 differentiation	Mouse lineage tracing and IRE1α perturbation; human transcriptional associations are not human fate tracing [34,37].
Monocyte-derived macrophages	Fibrogenic recruitment and matrix alteration	Phagocytosis and matrix processing	Mouse perturbation studies distinguish LGMN-associated matrix injury from CSF1-supported recruitment [50,51].
Cytotoxic lymphocytes	Impaired target recognition or inhibitory signals	Target-cell killing without epithelial or vascular injury	IPF spatial/cell studies and mouse checkpoint experiments [10]; cytotoxic T-cell evidence in SSc-ILD [53].
Vascular and lymphatic cells	Abnormal signaling and transport	Perfusion, barrier support and drainage	Mouse vascular perturbation and lymphatic tracer studies; vessel abundance does not establish perfusion or drainage [54,55].

**Table 2:** Experimental evidence and limitations of immune-directed treatments for pulmonary fibrosis.

Treatment and Target	Human Evidence and Setting	Model, Treatment Phase and Endpoint	Demonstrated Outcome	Interpretation and Safety Limits
MAF 116–124 (ALISNSHQL), APBB2 70–78 (YALTNIQAA) and TNS3 119–127 (SSYMHFTNV) peptide vaccines	IPF peptide profiling; healthy-donor MAF-specific, HLA-A*02:01-matched CTLs killed IPF myofibroblasts and IL-4-polarized macrophages in vitro	BLM mice: preventive priming, vaccine days 0/7/14 and BLM day 1; separate early post-injury MAF dosing days 7/14/21; endpoint day 28	Antigen-specific T-cell responses, lower collagen and fewer fibrosis-associated cells in mice; direct human target-cell killing shown separately in vitro	Day-7 dosing does not test persistent disease; HLA coverage, cross-reactivity and persistence of self-antigen immunity unresolved [15]
NKG2A blockade	IPF spatial/expression data; healthy-donor NK cytotoxicity; SSc-ILD PBMC activation and separate K562-target assay	BLM mice: delayed m20D5, 200 μg IV on days 14/17/20; endpoint day 24	Lower collagen and senescent-fibroblast abundance; no increase in the measured day-24 BAL inflammatory indices	Mouse antibody also targets NKG2C/E; no autologous IPF-fibroblast killing test; single-injury resolution and short safety follow-up limit translation [10]
CTLA4 blockade	IPF CTLA4-positive CD8-cell localization; human-CTLA4-knockin mice provide the intervention evidence	Repetitive BLM, 2 doses 14 days apart; delayed ipilimumab for 4.5 weeks. Results/legends start 21 days after dose 2; Methods give an unanchored “day 21”	Lower fibrosis/senescence signals, more proliferating AT2 cells and enhanced CD8 killing ex vivo; complete epithelial fate conversion not established	Schedule anchoring remains uncertain; other CTLA4-positive cells may contribute; oncology pulmonary toxicity cannot quantify an IPF treatment risk [13,123]
Transient FAP-CAR-T, CD5-targeted LNP-mRNA	Human IPF FAP-expression studies support target availability; therapeutic testing was in mice	Young/aged BLM mice: histological fibrosis evident by day 7; LNP-mRNA days 7/14; endpoint day 28 (early fibrotic phase)	Fewer FAP-positive fibroblasts, lower collagen/stiffness and altered epithelial states; RNA trajectories do not demonstrate lineage conversion	Single-injury early fibrotic disease; no matched duration comparison; repair after withdrawal and long-term normal-stromal safety remain untested [16]
FAP-CAR cytolytic Tregs	Human target-expression analysis; cell killing and treatment efficacy evaluated experimentally, not in patients	BLM mice: day-14 transfer beneficial; day-1 transfer ineffective/adverse on some collagen measures; additional day-28 transfer assessed on day 44	Fibroblast killing with immune suppression; lower collagen/histological fibrosis after day-14 or day-28 treatment; transferred cells detected at 1 month	Clonal/cell-sorting descriptions inconsistent; stability after withdrawal and later injury unresolved; murine cytokines do not establish human CRS safety [91]
Primary human FAP-CAR-NK	Primary human NK cells and human fibroblast/organoid cocultures; no treated IPF patients	BLM mice: feasibility experiment; 10 Gy TBI day 7, human-cell infusion 72 h later, endpoint 72 h after infusion; 2 tissue samples/group	Antigen-dependent killing in vitro; acute lung access and target engagement in vivo, without durable antifibrotic efficacy	High mortality, irradiation-conditioned hosts and no mock-CAR control; pulmonary benefit, persistence and systemic safety not established [90]
Hydrogel-primed FAP-CAR macrophages	Macrophage-cell-line functional assays; lung-treatment evidence mainly from mice	BLM mice: early post-injury cell infusion days 7/14; endpoint day 21	Fibroblast engulfment and collagen degradation in vitro; reduced mouse fibrosis; direct matrix degradation by transferred cells in vivo unresolved	Early fibrotic-phase treatment; persistent-disease efficacy, priming duration and effects on reparative stroma remain unresolved [89]
Myeloid FAP-CAR-knockin donor cells	No human pulmonary intervention	Lyz2-Cre FAP-CAR donors: prevention setting; donor macrophage/whole-blood transfer day 6 after BLM, recipient endpoint day 21	Donors resisted fibrosis induction; transferred cells reduced recipient fibrosis, without identifying which blood-cell component supplied the benefit	Early recipient treatment; mixed CAR-positive blood cells; duration, normal-tissue effects and clinical manufacturing/compatibility unresolved [122]
Anti-GD3 antibody	Human senescent-cell/NK assays	BLM mice: early anti-GD3 on days 5/8/11/14/17/20/23; endpoint day 27; paired μCT images at days 14/27	Improved NK degranulation, lower senescence-associated signals and collagen; lung target-cell death not directly established	Species-specific Siglec biology; day-14 μCT was after treatment began, not a pretreatment baseline; normal-tissue selectivity and durable safety unresolved [103]
CMA activation with CA77.1	IPF expression/lysosome assays; macrophage uptake mechanisms assessed in separate functional systems	BLM mice: early daily CA77.1 from day 2 or 7; endpoint day 15	Day-2 dosing reduced fibrosis more than day-7 dosing; separate assays supported macrophage uptake/efferocytosis mechanisms	Systemic CMA modulation; pulmonary benefit not exclusively assigned to efferocytosis or target-cell clearance; durable safety untested [115]
iNKT activation, α-galactosylceramide	Human senescent-cell/iNKT cocultures; no clinical pulmonary intervention	BLM mice: post-injury α-galactosylceramide day 10; cells assessed day 14, collagen day 21	Fewer senescence-associated cells, lower collagen and mortality; cytotoxic and cytokine-mediated effects not fully separated	Persistent-disease efficacy and target-cell fate not established; effector specificity and effects on normal repair remain unresolved [117]
FAP deficiency/FAP-positive cell depletion by CAR-T cells	Human FAP-expression studies; neither animal intervention supplies human treatment evidence	FAP loss: BLM/radiation models. Cell depletion: established repetitive BLM (first CAR-T 2 weeks after dose 6; repeat 2 weeks later) or Ad-TGF-β	FAP loss impaired collagen processing; CAR-T depletion worsened repetitive-BLM fibrosis; Ad-TGF-β histology changed little despite a small soluble-collagen increase	Enzyme loss, target-cell depletion and transient CAR expression are not matched interventions; exposure duration or stromal preservation cannot alone explain the differences [83,84]

BLM, bleomycin; BAL, bronchoalveolar lavage; CMA, chaperone-mediated autophagy; CRS, cytokine-release syndrome; IV, intravenous; LNP, lipid nanoparticle; PBMC, peripheral blood mononuclear cell; SSc-ILD, systemic sclerosis-associated interstitial lung disease; TBI, total body irradiation. Treatment phases describe the reported schedule, not proof of fibrosis at dosing: preventive starts before/around injury, early starts during acute injury/fibrogenesis, and delayed starts later. Established fibrosis requires evidence before treatment; persistence requires characterization over time. The CTLA4 timing discrepancy is an unanchored “day 21” in Methods versus 21 days after the second BLM dose in Results/legends (approximately day 35 after the first dose). The CAR-NK endpoint was 72 h after infusion, after a separate 72 h interval following irradiation; deaths/exclusions left 2 tissue samples/group. No study in this table demonstrates clinical efficacy in treated IPF patients.

**Table 3:** Required measurements for a clearance claim and additional evidence of lung repair.

Assessment Objective	Minimum Measurements or Controlled Comparisons	Interpretation
Target validity before clearance	Human target mapping in diseased, healthy and repairing cells; cell identity, anatomical distribution, persistence and functional perturbation	Shared markers may identify reparative cells; expression is a selection hypothesis, not proof that depletion is beneficial [11,12].
Essential evidence of target-cell elimination	Accessible antigen or peptide–HLA; target-specific death or observed engulfment with fate confirmation; independent cell counts/fate tracking; effector-dependence controls	Marker loss, altered secretion and cell contact alone do not establish physical elimination; in vitro killing does not establish lung clearance [15,74].
Stage-specific antifibrotic efficacy	Predose fibrosis and target burden; actual treatment schedule; histology plus collagen biochemistry; characterized persistence and complementary model	An early antifibrotic response may prevent fibrogenesis; a persistent-disease claim requires documented disease before treatment [18,146].
Explanation of conflicting outcomes	Hold target, model, pretreatment stage, delivered dose and effector program constant; vary exposure duration with longitudinal target-cell measurements	Current FAP studies cannot isolate exposure duration from targeting method, effector, stage or model [16,84].
Additional evidence of structural and cellular repair	Reparative-stromal survival; organized alveolar architecture/matrix; mature AT1 progeny with AT2 fate tracing where feasible; epithelial barrier and vascular/lymphatic performance	Proliferation or AT1-associated marker expression may indicate partial repair; cell lineage, morphology and functional interfaces require separate support [9,133,134].
Additional evidence of functional recovery and durability	Sustained lung mechanics or gas exchange; epithelial leak/perfusion measures; effector persistence after dosing; normal-tissue injury and repair after a later challenge	Structural and physiological responses can diverge; continued exposure may affect normal stroma and later repair [121,144].

## Data Availability

No new primary research data were generated or analyzed in this narrative review. The search strategies, selection criteria and reference-selection summary are provided in Supplementary Material S1.

## Supplementary Materials

The following supporting information can be downloaded at: https://www.mdpi.com/article/10.3390/biology15211991/s1, Supplementary Material S1: Table S1: Principal PubMed search strategies for the present review; Table S2: Criteria for relevance assessment and evidence selection; Table S3: Selection process and reference counts; Table S4: Individually retrieved supporting sources included in the initial reference collection.

## Abbreviations

AT1	alveolar type 1
AT2	alveolar type 2
BLM	bleomycin
CAR	chimeric antigen receptor
CAR-M	CAR macrophage
CMA	chaperone-mediated autophagy
CTL	cytotoxic T lymphocyte
ECM	extracellular matrix
FAP	fibroblast activation protein
HLA	human leukocyte antigen
ILD	interstitial lung disease
IPF	idiopathic pulmonary fibrosis
iNKT	invariant natural killer T
LNP	lipid nanoparticle
MHC	major histocompatibility complex
NK	natural killer
SSc-ILD	systemic sclerosis-associated interstitial lung disease
SASP	senescence-associated secretory phenotype
TGF-β	transforming growth factor-β
UIP	usual interstitial pneumonia
