# Supplementary material for: Selective Immune Clearance and Lung Repair in Pulmonary Fibrosis
Source: Biology (Basel). 2026 Nov 3;15(21):1991. doi: 10.3390/biology15211991 (PMC13761605; doi:10.3390/biology15211991)
Supplement: Supplementary file 1 [file biology-15-01991-s001.zip › biology-4660290-supplementary.pdf]

Supplementary Material S1

Literature Search and Study Selection

PubMed was systematically searched from May 2026 through 10 October 2026. Searches covered publications dated 1 January 1900 through 10 October 2026, with priority given to publications from 10 October 2024 through 10 October 2026. Primary searches used no language or species filter. Search results were ordered by publication date, newest first. Relevant mechanistic reports and foundational sources were also assessed for their contribution to the review.

1. Database search strategies

Table S1. Principal PubMed search strategies for the present review. Concept components are shown separately for clarity; the combined queries retain their original Boolean expressions and publication-date restrictions.

Step	Search strategy	Records
#1	Pulmonary fibrosis<br>("Pulmonary Fibrosis"[Mesh] OR "pulmonary fibrosis"[Title/Abstract] OR "lung fibrosis"[Title/Abstract] OR "idiopathic pulmonary fibrosis"[Title/Abstract])	Not separately counted
#2	Broad immune recognition and targeting<br>(immun*[Title/Abstract] OR senescen*[Title/Abstract] OR "natural killer"[Title/Abstract] OR "NK cell"[Title/Abstract] OR "antigen presentation"[Title/Abstract] OR "CAR-T"[Title/Abstract] OR "CAR T"[Title/Abstract] OR "CAR-M"[Title/Abstract] OR "chimeric antigen receptor"[Title/Abstract] OR CTLA4[Title/Abstract] OR "CTLA-4"[Title/Abstract] OR "HLA-E"[Title/Abstract] OR NKG2A[Title/Abstract] OR "FAP"[Title/Abstract])	Not separately counted
#3	Senescence and epithelial repair<br>(senescen*[Title/Abstract] OR transitional[Title/Abstract] OR "alveolar regeneration"[Title/Abstract] OR "alveolar repair"[Title/Abstract] OR "KRT8"[Title/Abstract] OR "KRT17"[Title/Abstract])	Not separately counted
#4	Matrix mechanics and metabolic support<br>(mechanotransduction[Title/Abstract] OR stiffness[Title/Abstract] OR "mechanical memory"[Title/Abstract] OR YAP[Title/Abstract] OR TAZ[Title/Abstract] OR "immunometabolism"[Title/Abstract] OR "lipid metabolism"[Title/Abstract] OR "metabolic reprogramming"[Title/Abstract])	Not separately counted
#5	Focused immune recognition and clearance<br>("immune clearance"[Title/Abstract] OR "immune surveillance"[Title/Abstract] OR immunosurveillance[Title/Abstract] OR "natural killer"[Title/Abstract] OR "NK cells"[Title/Abstract] OR "antigen presentation"[Title/Abstract] OR immunopeptidom*[Title/Abstract] OR "chimeric antigen receptor"[Title/Abstract] OR "CAR-T"[Title/Abstract] OR "CAR T"[Title/Abstract] OR "CAR-M"[Title/Abstract] OR CTLA4[Title/Abstract] OR "CTLA-4"[Title/Abstract] OR "HLA-E"[Title/Abstract] OR NKG2A[Title/Abstract] OR "CD47"[Title/Abstract] OR "GD3"[Title/Abstract] OR "cytotoxic T"[Title/Abstract] OR "fibroblast activation protein"[Title/Abstract])	Not separately counted

Step	Search strategy	Records
#6	Senescence and immune recognition across organs<br>(senescen*[Title/Abstract] AND ("immune clearance"[Title/Abstract] OR "immune surveillance"[Title/Abstract] OR immunosurveillance[Title/Abstract] OR "natural killer"[Title/Abstract] OR "chimeric antigen receptor"[Title/Abstract] OR "CAR T"[Title/Abstract] OR "CAR-T"[Title/Abstract] OR NKG2A[Title/Abstract] OR "HLA-E"[Title/Abstract] OR "antigen presentation"[Title/Abstract]))	Not separately counted
#7	Broad pulmonary immune-targeting search<br>(("Pulmonary Fibrosis"[Mesh] OR "pulmonary fibrosis"[Title/Abstract] OR "lung fibrosis"[Title/Abstract] OR "idiopathic pulmonary fibrosis"[Title/Abstract]) AND (immun*[Title/Abstract] OR senescen*[Title/Abstract] OR "natural killer"[Title/Abstract] OR "NK cell"[Title/Abstract] OR "antigen presentation"[Title/Abstract] OR "CAR-T"[Title/Abstract] OR "CAR T"[Title/Abstract] OR "CAR-M"[Title/Abstract] OR "chimeric antigen receptor"[Title/Abstract] OR CTLA4[Title/Abstract] OR "CTLA-4"[Title/Abstract] OR "HLA-E"[Title/Abstract] OR NKG2A[Title/Abstract] OR "FAP"[Title/Abstract])) AND ("1900/01/01"[Date - Publication] : "2026/10/10"[Date - Publication])	9,863 identified; 2,800 retrieved
#8	Senescence, transitional epithelium and alveolar repair<br>(("Pulmonary Fibrosis"[Mesh] OR "pulmonary fibrosis"[Title/Abstract] OR "lung fibrosis"[Title/Abstract] OR "idiopathic pulmonary fibrosis"[Title/Abstract]) AND (senescen*[Title/Abstract] OR transitional[Title/Abstract] OR "alveolar regeneration"[Title/Abstract] OR "alveolar repair"[Title/Abstract] OR "KRT8"[Title/Abstract] OR "KRT17"[Title/Abstract])) AND ("1900/01/01"[Date - Publication] : "2026/10/10"[Date - Publication])	1,015 identified; 1,015 retrieved
#9	Matrix mechanics and related mechanisms<br>(("Pulmonary Fibrosis"[Mesh] OR "pulmonary fibrosis"[Title/Abstract] OR "lung fibrosis"[Title/Abstract] OR "idiopathic pulmonary fibrosis"[Title/Abstract]) AND (mechanotransduction[Title/Abstract] OR stiffness[Title/Abstract] OR "mechanical memory"[Title/Abstract] OR YAP[Title/Abstract] OR TAZ[Title/Abstract] OR "immunometabolism"[Title/Abstract] OR "lipid metabolism"[Title/Abstract] OR "metabolic reprogramming"[Title/Abstract])) AND ("1900/01/01"[Date - Publication] : "2026/10/10"[Date - Publication])	711 identified; 711 retrieved
#10	Focused immune recognition and clearance<br>(("Pulmonary Fibrosis"[Mesh] OR "pulmonary fibrosis"[Title/Abstract] OR "lung fibrosis"[Title/Abstract] OR "idiopathic pulmonary fibrosis"[Title/Abstract]) AND ("immune clearance"[Title/Abstract] OR "immune surveillance"[Title/Abstract] OR immunosurveillance[Title/Abstract] OR "natural killer"[Title/Abstract] OR "NK cells"[Title/Abstract] OR "antigen presentation"[Title/Abstract] OR immunopeptidom*[Title/Abstract] OR "chimeric antigen receptor"[Title/Abstract] OR "CAR-T"[Title/Abstract] OR "CAR T"[Title/Abstract] OR "CAR-M"[Title/Abstract] OR CTLA4[Title/Abstract] OR "CTLA-4"[Title/Abstract] OR "HLA-E"[Title/Abstract] OR NKG2A[Title/Abstract] OR "CD47"[Title/Abstract] OR "GD3"[Title/Abstract] OR "cytotoxic T"[Title/Abstract] OR "fibroblast activation protein"[Title/Abstract])) AND ("1900/01/01"[Date - Publication] : "2026/10/10"[Date - Publication])	344 identified; 344 retrieved

Step	Search strategy	Records
#11	Mechanistic support across organs<br>(senescen*[Title/Abstract] AND ("immune clearance"[Title/Abstract] OR "immune surveillance"[Title/Abstract] OR immunosurveillance[Title/Abstract] OR "natural killer"[Title/Abstract] OR "chimeric antigen receptor"[Title/Abstract] OR "CAR T"[Title/Abstract] OR "CAR-T"[Title/Abstract] OR NKG2A[Title/Abstract] OR "HLA-E"[Title/Abstract] OR "antigen presentation"[Title/Abstract])) AND ("1900/01/01"[Date - Publication] : "2026/10/10"[Date - Publication])	1,031 identified; 800 retrieved
#12	Focused immune recognition, recent publications<br>(("Pulmonary Fibrosis"[Mesh] OR "pulmonary fibrosis"[Title/Abstract] OR "lung fibrosis"[Title/Abstract] OR "idiopathic pulmonary fibrosis"[Title/Abstract]) AND ("immune clearance"[Title/Abstract] OR "immune surveillance"[Title/Abstract] OR immunosurveillance[Title/Abstract] OR "natural killer"[Title/Abstract] OR "NK cells"[Title/Abstract] OR "antigen presentation"[Title/Abstract] OR immunopeptidom*[Title/Abstract] OR "chimeric antigen receptor"[Title/Abstract] OR "CAR-T"[Title/Abstract] OR "CAR T"[Title/Abstract] OR "CAR-M"[Title/Abstract] OR CTLA4[Title/Abstract] OR "CTLA-4"[Title/Abstract] OR "HLA-E"[Title/Abstract] OR NKG2A[Title/Abstract] OR "CD47"[Title/Abstract] OR "GD3"[Title/Abstract] OR "cytotoxic T"[Title/Abstract] OR "fibroblast activation protein"[Title/Abstract])) AND ("2024/10/10"[Date - Publication] : "2026/10/10"[Date - Publication])	100 identified; count only
#13	Focused immune recognition, review publications<br>(("Pulmonary Fibrosis"[Mesh] OR "pulmonary fibrosis"[Title/Abstract] OR "lung fibrosis"[Title/Abstract] OR "idiopathic pulmonary fibrosis"[Title/Abstract]) AND ("immune clearance"[Title/Abstract] OR "immune surveillance"[Title/Abstract] OR immunosurveillance[Title/Abstract] OR "natural killer"[Title/Abstract] OR "NK cells"[Title/Abstract] OR "antigen presentation"[Title/Abstract] OR immunopeptidom*[Title/Abstract] OR "chimeric antigen receptor"[Title/Abstract] OR "CAR-T"[Title/Abstract] OR "CAR T"[Title/Abstract] OR "CAR-M"[Title/Abstract] OR CTLA4[Title/Abstract] OR "CTLA-4"[Title/Abstract] OR "HLA-E"[Title/Abstract] OR NKG2A[Title/Abstract] OR "CD47"[Title/Abstract] OR "GD3"[Title/Abstract] OR "cytotoxic T"[Title/Abstract] OR "fibroblast activation protein"[Title/Abstract])) AND ("1900/01/01"[Date - Publication] : "2026/10/10"[Date - Publication]) AND (Review[Publication Type] OR "Systematic Review"[Publication Type] OR "Meta-Analysis"[Publication Type])	74 identified; count only

Counts reflect the search results as of 10 October 2026. “Records” indicates the total number of results for each search, whereas “Retrieved” indicates the number obtained for relevance screening. Individual search components were not run separately, so separate counts are unavailable. Searches limited to recent publications or reviews were used only to obtain counts and did not add records for screening. Because the searches overlap, their counts should not be added together. The full search strings retain the original publication-date limits.

2. Eligibility and selection priorities

Table S2. Criteria for relevance assessment and evidence selection.

Criterion	Application
Scope	Immune recognition or targeting of fibrosis-associated cells; preservation of pulmonary repair; matrix or epithelial mechanisms needed to interpret these processes.

Criterion	Application
Eligible evidence	Human tissue studies, functional human-cell experiments, animal interventions, lineage-tracing studies, relevant clinical trials and methodological guidance.
Priority for primary studies	Defined targets or effectors, causal perturbations, treatment timing, adverse or contradictory results, and measurements of repair or physiological function.
Earlier literature	Foundational mechanistic experiments, major counterexamples, lineage-tracing evidence and clinical safety studies were retained regardless of the two-year priority window.
Cross-organ evidence	Included only when informative for a recognition, effector or repair mechanism; the source disease, organ and model were stated.
Reviews and guidelines	Used for background, reference tracing and accepted definitions; they did not substitute for original experiments when interpreting experimental results.
Lower-priority reports	Reports without a relevant targeting or repair mechanism, additional overlapping descriptive studies and redundant background reviews were not prioritized for citation.
Full-text assessment	Relevant methods, results, figures and available supplements were examined for source cells, species, target identity, treatment sequence, comparison groups and outcomes.
Evidence synthesis	Qualitative comparison of association/target expression, recognition/modulation, physical elimination and tissue or physiological recovery; no pooled effect estimates or formal risk-of-bias score.

3. Screening and reference selection

Table S3. Selection process and reference counts.

Stage	Procedure	Available count
1. Identification of relevant mechanistic and foundational sources	Relevant foundational and mechanistic reports were identified through targeted source checking and assessed for their contribution to fibrosis, immune targeting or tissue repair.	Five individually retrieved supporting sources are listed in Table S4; no separate total of targeted source checks was recorded.
2. Pulmonary immune-targeting and supporting searches	Five searches addressed pulmonary immune targeting, senescence and repair, matrix mechanics, focused recognition/clearance and cross-organ mechanistic support.	Individual yields and retrieval limits are shown in Table S1; overlapping queries were not added together.
3. Relevance assessment and reference collection	Titles and abstracts were assessed against the review scope. Duplicate records were identified by PMID and bibliographic relationships.	191 unique records in the initial reference collection, assembled from literature searches and assessment of supporting sources.
4. Detailed assessment	Potentially informative original reports were examined in full text and, when available, with their supplements. Mechanistic, comparative and contradictory evidence received priority.	139 records from the initial collection appear in the final bibliography; 52 were not selected for citation.
5. Additional identification	Relevant sources were added through targeted assessment and reference tracing.	16 additional cited sources. The individual identification route was not recorded for every added source.
6. Final synthesis	Evidence was integrated by cell state, recognition route, intervention phase and repair outcome.	155 numbered references. This is the final bibliography count.

Retrieval limits and qualitative selection may have omitted relevant publications. Neither a full-text exclusion tally nor study-level exclusion reasons were recorded.

4. Targeted retrieval of foundational sources

Table S4. Individually retrieved supporting sources included in the initial reference collection.

Source	Reason retained
[11] PMID: 36227993<br>Reyes, N.S.; Krasilnikov, M.; Allen, N.C.; Lee, J.Y.; Hyams, B.; Zhou, M.; Ravishankar, S.; Cassandras, M.; Wang, C.; Khan, I.; et al. Sentinel p16INK4a+ cells in the basement membrane form a reparative niche in the lung. Science 2022, 378, 192–201.	Reparative functions of p16-positive stromal cells; qualification of senescence-directed targeting.
[12] PMID: 39121846<br>Ogrodnik, M.; Carlos Acosta, J.; Adams, P.D.; d'Adda di Fagagna, F.; Baker, D.J.; Bishop, C.L.; Chandra, T.; Collado, M.; Gil, J.; Gorgoulis, V.; et al. Guidelines for minimal information on cellular senescence experimentation in vivo. Cell 2024, 187, 4150–4175.	Criteria for interpreting and reporting cellular senescence in vivo.
[119] PMID: 31511695<br>Aghajanian, H.; Kimura, T.; Rurik, J.G.; Hancock, A.S.; Leibowitz, M.S.; Li, L.; Scholler, J.; Monslow, J.; Lo, A.; Han, W.; et al. Targeting cardiac fibrosis with engineered T cells. Nature 2019, 573, 430–433.	Foundational FAP-directed CAR-T targeting of cardiac fibrosis; cross-organ supporting evidence.
[120] PMID: 34990237<br>Rurik, J.G.; Tombácz, I.; Yadegari, A.; Méndez Fernández, P.O.; Shewale, S.V.; Li, L.; Kimura, T.; Soliman, O.Y.; Papp, T.E.; Tam, Y.K.; et al. CAR T cells produced in vivo to treat cardiac injury. Science 2022, 375, 91–96.	Transient in vivo CAR-T generation using mRNA delivery; cross-organ platform evidence.
[121] PMID: 23712432<br>Tran, E.; Chinnasamy, D.; Yu, Z.; Morgan, R.A.; Lee, C.C.; Restifo, N.P.; Rosenberg, S.A. Immune targeting of fibroblast activation protein triggers recognition of multipotent bone marrow stromal cells and cachexia. J. Exp. Med. 2013, 210, 1125–1135.	Recognition of normal FAP-expressing stromal cells and systemic toxicity.
